# Research progress on the correlation between gut microbiota and the occurrence of hyperuricemia

**DOI:** 10.3389/fmicb.2026.1763753

**Published:** 2026-05-04

**Authors:** Qiaoyi Wang, Li Yan, Yufeng Mo, Yuzhu Dai, Jun Cheng, Minli Qiu

**Affiliations:** 1 Department of Clinical Laboratory, The 903rd Hospital of the Joint Logistic Support Force, Hangzhou, China; 2 Department of Health Economic, The 903rd Hospital of the Joint Logistic Support Force, Hangzhou, China

**Keywords:** gut microbiota, hyperuricemia, lipopolysaccharide, short chain fatty acids, uric acid

## Abstract

Hyperuricemia is a metabolic disorder caused by excessive uric acid production or reduced uric acid excretion in the body. Approximately 30% of the uric acid in the human body is excreted through the intestines. As a vital component of the intestine, the gut microbiota influences host uric acid levels through multiple mechanisms and pathways, including purine metabolism, short chain fatty acid production, and intestinal barrier function. Additionally, hyperuricemia can also affect the distribution of gut microbiota, and a dysbiosis in this distribution may further trigger inflammation. This paper reviews the correlation between gut microbiota and the occurrence of hyperuricemia, providing a reference for treating hyperuricemia through the gut microbiota.

## 1 Introduction

The prevalence of hyperuricemia (HUA) has been increasing year by year, becoming a major global public health issue. Data indicated that the prevalence rates of HUA among American men and women reached as high as 20.2% and 20.0%, respectively (Chen-Xu et al., 2019). In South Korea, the prevalence rate of HUA in the general population was as high as 11.4% (Kim et al., 2018), while in Australia, it reached 16.6% (Ting et al., 2016). Furthermore, between 2000 and 2017, the prevalence of HUA in the Chinese population sharply increased from 8.5% to 18.4% (Li et al., 2021), with patients showing a trend toward younger age (Zhang et al., 2021), making it the second most common metabolic disorder after type 2 diabetes (Zhao R. et al., 2022). HUA is a metabolic disorder caused by increased uric acid production or reduced uric acid excretion due to abnormal purine metabolism. It is characterized by fasting serum uric acid levels exceeding 420 μmol/L (360 μmol/L for females) on two separate occasions under a standard purine diet (Li et al., 2021). Most patients exhibit unhealthy dietary habits such as drinking alcohol, smoking, and long-term intake of high-fructose, high-fat, and high-purine diets, as well as unhealthy lifestyles characterized by prolonged sitting, lack of physical exercise, and obesity (Zhang et al., 2021; Long and Liu, 2022; He et al., 2022). One study reported that when serum uric acid levels exceeded 535 μmol/L in men and 416 μmol/L in women, median life expectancy decreased by 11.7 and 5.9 years, respectively (Browne et al., 2021). The effects of HUA extend beyond gout, it is also a significant independent risk factor for hypertension (Han et al., 2021), kidney disease (Jung et al., 2020), cardiovascular disease (Li et al., 2024), and diabetes (Su et al., 2021). Furthermore, some studies have indicated that long-term HUA increases mortality rates among patients with hypertension (Yin et al., 2024), chronic heart failure (Xie et al., 2024), acute respiratory distress syndrome (Lee et al., 2019), and critically ill patients with acute kidney injury (Hu et al., 2025).

Uric acid is primarily excreted through the kidneys in urine, with approximately 30% excreted through the intestines (Lv et al., 2020), forming a complex intestinal-renal axis regulatory network (Chen et al., 2019). Uric acid is excreted through the intestines in two ways. One way is the direct excretion of the original form. The uric acid in the blood is transported by transport proteins or undergoes passive diffusion into the intestinal lumen, and then exits the body as the original form in the feces. The other way is the bacterial decomposition metabolism. The uric acid in the blood is decomposed by the uricase produced by the gut microbiota in the large intestine into smaller water-soluble molecules such as ammonia, carbon dioxide and water, and is ultimately metabolized and excreted. The gut microbiota is a vast and complex community consisting of over 100 trillion microbial cells, which are colonized in the intestines and play roles in immune regulation, barrier protection, and metabolic functions. The human gut microbiota primarily consists of six major bacterial phyla: Firmicutes, Bacteroidetes, Proteobacteria, Actinobacteria, Verrucomicrobia, and Fusobacteria. Among these, Firmicutes and Bacteroidetes account for 90% of the total (Cresci, 2025). A growing body of research indicates that the gut microbiota and its metabolites play a significant role in the occurrence and development of HUA (Wang et al., 2022). The specific mechanisms of action are summarized in Table 1. To this end, this paper reviews the latest clinical research advances over the past decade regarding the correlation between gut microbiota and HUA. It explores the mechanisms by which gut microbiota regulate uric acid metabolism, including their involvement in purine metabolism and uric acid production, as well as the influence of gut microbial metabolites on uric acid levels. Furthermore, it elucidates that HUA triggers dysbiosis in gut microbiota distribution. And it elaborates on the mechanism by which this dysbiosis induces the occurrence of inflammation. Finally, it envisions that targeted gut microbiota therapy may emerge as a novel approach for the prevention and treatment of HUA.

**TABLE 1:** Mechanisms of action of major gut microbiota associated with hyperuricemia.

Effects	Bacterial phyla	Bacterial genus	Mechanisms of action	References
Positive effect	Actinobacteria	*Bifidobacterium*	Promote purine metabolism, reduce absorption of uric acid precursors, anti-inflammatory	Liu P. et al., 2025; Lu et al., 2020
	Verrucomicrobia	*Akkermansia*	Associated with amino acid levels, regulates intestinal ABCG2 and renal ABCG2, URAT1, and GLUT9, repairs the intestinal barrier	Song et al., 2022; Lv et al., 2023; Zhang et al., 2022
	Firmicutes	*Blautia*	Produce SCFAs	Liang et al., 2022
		*Lachnospira*	Produces butyrate, anti-inflammatory, repairs the intestinal barrier	Liang et al., 2022; Mendez-Salazar et al., 2021
		*Ruminococcus* (*Coprococcus*)	Produces butyrate, anti-inflammatory, repairs the intestinal barrier	Liang et al., 2022; Mendez-Salazar et al., 2021
		*Butyricicoccus*	Produces butyrate, anti-inflammatory, repairs the intestinal barrier	Liang et al., 2022; Mendez-Salazar et al., 2021
		*Lactobacilli*	Inhibits XO activity, breaks down purines, synthesizes uricase, anti-inflammatory	Singh et al., 2024; Yamada et al., 2016; Kuo et al., 2021; Lu et al., 2020
Bidirectional effect	Bacteroidetes	*Bacteroides* (positive effect)	Upregulate ABCG2 expression, downregulate xanthine dehydrogenase expression, repair the intestinal barrier	Song et al., 2023; Lv et al., 2023
		*Bacteroides* (negative effect)	Pro-inflammatory, altering the ratio of the microbiota to cause dysbacteriosis	Guo et al., 2016, 2021; Chu et al., 2021; Kim et al., 2022
	Proteobacteria	*Escherichia coli* (positive effect)	Convert uric acid into SCFAs, break down purines via the 2,8-dihydropurine pathway, break down uric acid	Liu Y. et al., 2023, 2025
		*Escherichia coli* (negative effect)	Secretes xanthine dehydrogenase, promoting uric acid production, pro-inflammatory	Singh et al., 2024
Negative effect	Proteobacteria	*Proteus*	Secretes xanthine dehydrogenase, promoting uric acid production, pro-inflammatory	Singh et al., 2024
		*Shigella*	Promotes inflammation, disrupts the intestinal barrier	Liang et al., 2022
	Fusobacteria	*Fusobacterium*	Promotes inflammation, disrupts the intestinal barrier	Chu et al., 2021
	Bacteroidetes	*Prevotella*	Promotes inflammation, disrupts the intestinal barrier	Chu et al., 2021

Positive effect indicates the ability to inhibit the increase of uric acid levels. Negative effect indicates the ability to promote the increase of uric acid levels. Bidirectional effect indicates both the ability to inhibit the increase of uric acid levels and the ability to promote the increase of uric acid levels.

## 2 Gut microbiota participates in purine metabolism and uric acid production

**FIGURE 1:**
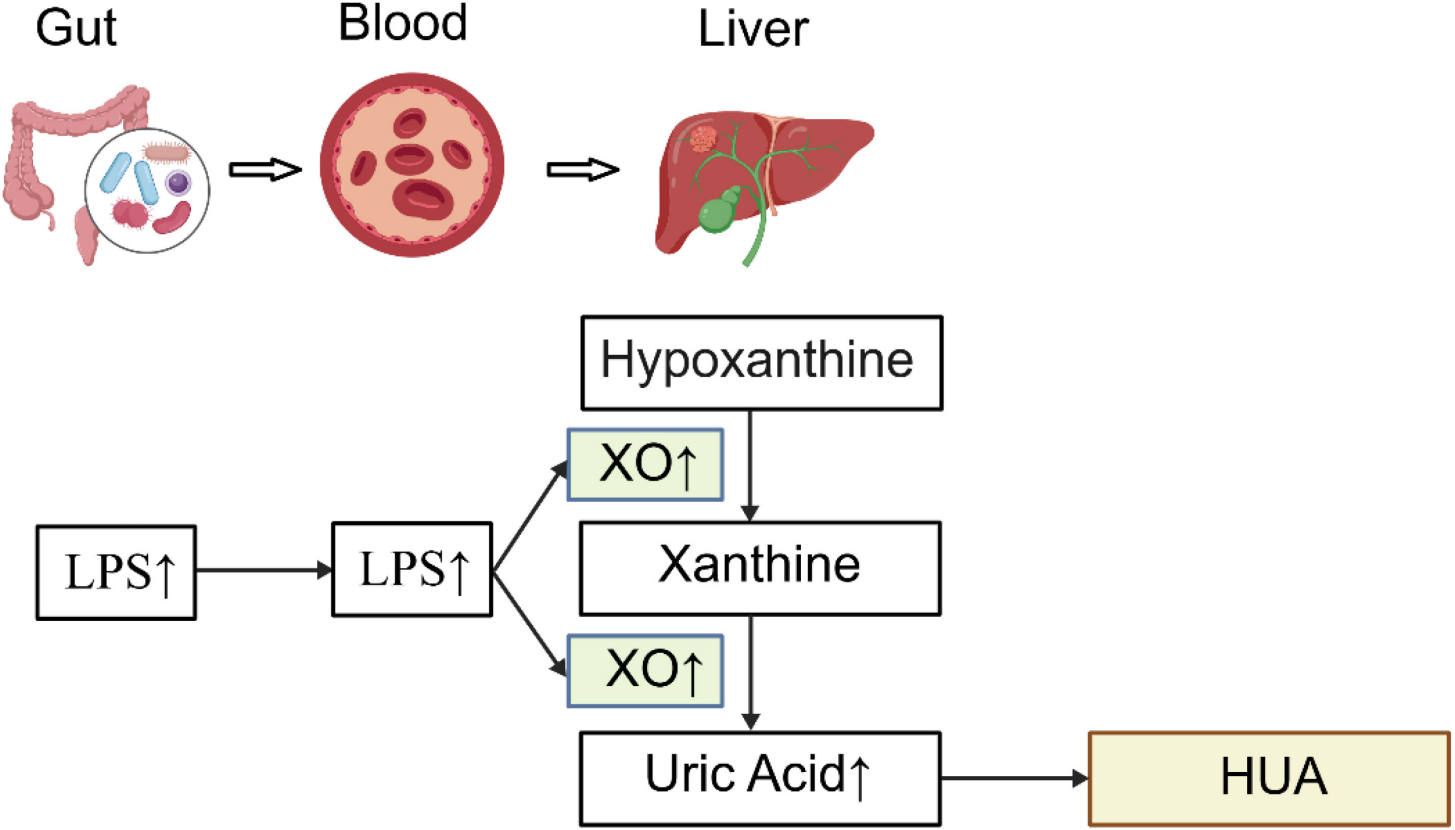
The mechanistic link between LPS, XO, and uric acid production. LPS produced by the gut microbiota enters the bloodstream, and upon reaching the liver, upregulates the expression of XO. XO then catalyzes the conversion of hypoxanthine to xanthine, and subsequently xanthine to uric acid, thereby promoting uric acid production and ultimately leading to HUA. Created with BioGDP.com (Jiang S. et al., 2025).

Uric acid is the end product of purine metabolism in the human body. Adenine nucleotides and guanine nucleotides undergo steps including deamination and dephosphorylation of ribose. Ultimately, under the catalysis of xanthine oxidase (XO), hypoxanthine is oxidized to form xanthine, which is then further oxidized to form uric acid (Maiuolo et al., 2016). Excessive uric acid production from purine degradation in the liver is the primary cause of HUA. Xanthine oxidoreductase is a rate-limiting enzyme composed of two distinct forms: xanthine dehydrogenase and XO. Its activity may represent the true risk factor for HUA. Inhibiting the activity of xanthine oxidoreductase may alleviate oxidative stress, increase ATP production, and reduce uric acid levels (Furuhashi, 2020). XO is a key enzyme in purine metabolism, primarily catalyzing the conversion of purine to uric acid. Lipopolysaccharide (LPS), a component of the cell walls of Gram-negative bacteria in the gut microbiota, are one of the primary factors regulating XO activity. LPS upregulates the activity of XO, generating reactive oxygen species (ROS), which directly activates the NOD-like receptor family pyrin domain containing 3 (NLRP3) inflammasome (Wu et al., 2020). The mechanistic link between LPS, XO, and uric acid production is shown in Figure 1. Animal studies further confirmed that compared to normal mice, HUA mice exhibited significantly reduced levels of Bifidobacteria and Lactobacilli in their feces, alongside markedly elevated LPS levels, XO activity and uric acid levels (Lu et al., 2020). Bifidobacteria and Lactobacilli exert inhibitory effects on the formation of uric acid. They competitively utilize purines with the host, effectively reducing the total amount of purines available for absorption by the host. Studies have shown that when the L. gasseri strain PA-3 is consumed along with food in the human body, it can effectively reduce the absorption of purines by the intestines (Yamada et al., 2016). *Lactobacillus reuteri* TSR332 and *Lactobacillus fermentum* TSF331 can eliminate excess purine assimilation in the digestive tract by degrading purine nucleosides (Kuo et al., 2021). Bifidobacteria express purine nucleotide phosphorylase, promoting intestinal purine metabolism and reducing the absorption of uric acid precursors (Liu P. et al., 2025). Conversely, Escherichia coli and Proteus promote uric acid production by secreting xanthine dehydrogenase, which catalyzes the conversion of hypoxanthine and xanthine into uric acid (Singh et al., 2024). Therefore, the gut microbiota plays a crucial role in regulating purine and uric acid metabolism.

## 3 Gut microbiota metabolites influence uric acid levels

The gut microbiota converts dietary components and endogenous substances within the host into important bioactive molecules through complex metabolic activities. The primary metabolites include short chain fatty acids (SCFAs) produced by the fermentation of dietary fiber, essential amino acids derived from protein breakdown, and certain compounds synthesized by the bacteria themselves, such as uricase involved in purine metabolism and LPS that induces inflammation. The specific mechanisms by which these metabolites regulate uric acid levels will now be elaborated in detail from the following aspects.

### 3.1 Uricase

In most mammals, uric acid is converted into 5-hydroxyisouric acid by uricase, then spontaneously decomposes into the more soluble allantoin, and is ultimately excreted through the kidneys. However, during the early stages of human evolution, a mutation occurred in the uricase gene, resulting in the complete loss of uric acid metabolism function. As a result, uric acid could not be broken down into the more water-soluble allantoin (Kratzer et al., 2014). Some bacteria that are colonized in the intestines can break down a portion of the uric acid produced daily, such as *Lactobacillus* and *Pseudomonas* (Singh et al., 2024). These bacteria can synthesize uricase, thereby reducing the absorption of purines and effectively lowering the risk of uric acid accumulation in the body. In recent years, significant progress has been made in this field. Researchers discovered a widely distributed gene cluster in the gut microbiota encoding a uric acid degradation pathway. This pathway utilizes purines or uric acid as carbon sources and energy sources for anaerobic metabolism (Kasahara et al., 2023). Under anaerobic conditions, it can convert uric acid into SCFAs. For example, *Escherichia coli* transforms uric acid into acetate under anaerobic conditions (Liu Y. et al., 2023), thereby compensating for the deficiency of uricase. The latest research indicated that Clostridium sporogenes and *E. coli* may achieve anaerobic degradation of purines through the 2,8-dioxopurine pathway (Liu Y. et al., 2025). Therefore, the anaerobic gut microbiota plays a crucial role in compensating for uricase in the human body. The use of antibiotics targeting the activity of anaerobic bacteria may increase the risk of HUA in humans. It is recommended to exercise caution when prescribing anaerobic antibiotics to HUA patients (Wang and Ye, 2024).

### 3.2 Amino acids

Amino acid metabolism disorders are one of the common metabolic characteristics of HUA. Some studies suggest that in HUA, dysbiosis may be accompanied by amino acid metabolism disorders. Research combining metabolomics and gut microbiota diversity analysis revealed that HUA patients exhibited downregulated expression of serine, glutamic acid, and glutamine, alongside dysbiosis characterized by a significant increase in opportunistic pathogens and a marked reduction in SCFA-producing bacteria (Pan et al., 2020). Another study comparing uricase knockout mice with control mice revealed significant alterations in fecal metabolites, most notably in branched-chain amino acids including leucine, isoleucine, and valine. Their levels were closely associated with certain bacteria such as *Akkermansia*, which were markedly reduced in HUA model mice (Song et al., 2022). Some studies have revealed a significant correlation between the gut microbiota and amino acid metabolism in HUA. Through correlation analysis, one study found that *Akkermansia*, UCG-005, Lachnospiridae_NK4A136_group, Lactococcus, and Butyromonas were associated with the levels of various amino acids (Liu P. et al., 2024). Another study found that HUA rats exhibited a significant decrease in gut microbiota diversity and a significant increase in serum levels of amino acids such as leucine, phenylalanine, and tyrosine. Furthermore, correlation analysis further revealed a significant association between Bacteroidetes and the levels of these amino acids (Ma et al., 2026). However, current evidence is limited to correlational studies, and the regulatory mechanisms underlying the relationship between the two in HUA remain unclear. It is possible that certain amino acids cannot be synthesized endogenously and must rely on fermentation by the gut microbiota. Alternatively, it may be that amino acid metabolism disorders alter the intestinal microenvironment, thereby reshaping the gut microbiota structure. This requires further research and clarification.

### 3.3 Butyric acid

The most abundant SCFAs in the intestine are acetate, propionate, and butyrate, which participate in immune function, apoptosis, lipid metabolism, and inflammation (Zhang et al., 2023). There are relatively few studies on the effects of acetate and propionate on HUA, and a few studies have shown that acetate can treat HUA by reducing XO activity and uric acid levels (Oyabambi et al., 2020). Among SCFAs, butyrate has been the most extensively studied. Produced by the fermentation of dietary fiber by gut bacteria, it serves as the primary energy source for the intestine and regulates uric acid metabolism through multiple pathways. On one hand, butyrate enhances the expression of intestinal epithelial tight junction proteins Occludin and ZO-1, thereby protecting the intestinal barrier, reversing elevated levels of inflammatory factors, and reducing serum uric acid levels (Li et al., 2023; Peng et al., 2009). On the other hand, butyrate significantly upregulates the expression of the ATP-binding cassette transporter G2 (ABCG2), a key efflux transporter regulating uric acid excretion, thereby promoting active intestinal excretion of uric acid (Wang and Ye, 2024). However, the mechanism by which butyrate modulates HUA remains unclear. Research by Gao et al. (2023) indicated that butyrate alleviated intestinal inflammation by reducing the expression of IL-6 and IL-1β while increasing the expression of IL-10. Guo et al. (2021) demonstrated through experiments using inulin to alleviate HUA that butyrate exhibited negative correlations with inflammatory factors tumor necrosis factor-α (TNF-α), IL-1β, and IL-6, as well as with LPS and uric acid levels. This indicates that butyric acid may intervene in the occurrence and development of HUA by regulating targets such as IL-1β and IL-6.

ABCG2 is located on the apical membrane of intestinal epithelial cells and is responsible for actively transporting uric acid from within intestinal epithelial cells into the intestinal lumen. Its expression in dendritic cells is mediated by activation of the peroxisome proliferator-activated receptor gamma (PPARγ) (Szatmari et al., 2006). Dysfunction of ABCG2 readily leads to insufficient uric acid excretion and induces HUA. Both Xie et al. (2021), Yang et al. (2025) have demonstrated through animal studies that butyrate, a gut microbiota metabolite, may influence uric acid excretion by regulating ABCG2 expression through activation of the PPARγ signaling pathway. It is worth noting that the effects of butyrate are highly concentration-dependent. In terms of immune regulation, butyrate at low concentrations (0.1 mM) exerts an anti-inflammatory effect by inhibiting TNF-α production through the PPARγ signaling pathway; conversely, butyrate at high concentrations (10 mM) promotes macrophage death via the G protein-coupled receptor pathway and induces the production of IL-1β and TNF-α, thereby exerting a pro-inflammatory effect (Jiang M. et al., 2025). Regarding intestinal barrier function, butyrate promotes intestinal barrier function at low concentrations (≤2 mM), but may impair it at high concentrations (5 or 8 mM) by inducing apoptosis (Liu et al., 2018). This “double-edged sword” characteristic suggests that the uric acid-lowering effect of butyrate may be significantly influenced by concentration. Therefore, identifying the optimal concentration range for butyrate to exert its uric acid-lowering effect is of great significance for clinical translation. Another important uric acid transporter is solute carrier protein 2 family 9 (SLC2A9), which plays a key role in uric acid reabsorption. Wu et al. (2023) determined through polymorphism studies that the SLC2A9 mutation site rs16890979 may reduce renal uric acid reabsorption. Ying et al. (2024) established a HUA animal model and concluded that gut microbiota in HUA patients may increase intestinal uric acid production by enhancing XO activity and SLC2A9 uric acid reabsorption capacity. However, whether the gut microbiota and its metabolites can remotely regulate the activity of renal uric acid reabsorption transporters URAT1 and GLUT9 or the expression of the ABCG2 protein in the kidney via the “gut-kidney axis,” thereby indirectly affecting renal uric acid processing capacity, remains to be further elucidated. Zhang et al. (2022) found that *Akkermansia muciniphila* can inhibit urate reabsorption by reducing the expression of URAT1 and GLUT9 in the kidneys. Additionally, a study isolated a strain of Bacteroides xylanisolvens from human feces. By establishing a model of HUA in goslings, it was discovered that Bacteroides xylanisolvens not only downregulated XDH mRNA expression in the liver but also upregulated ABCG2 mRNA expression in the kidneys (Song et al., 2023).

## 4 Hyperuricemia triggers dysbiosis in gut microbiota distribution

### 4.1 Hyperuricemia affects the distribution of gut microbiota

Multiple clinical studies and animal experiments indicate significant differences in gut microbiota distribution between patients with HUA and healthy controls. Serum uric acid levels are inversely correlated with gut microbiota diversity (Ness et al., 2025). Liu et al. (2020) established a high-purine diet-induced HUA rat model and found that the genera *Vallitalea*, *Christensenella*, and *Insolitispirillum* were associated with HUA. In the gut microbiota of HUA patients, certain opportunistic pathogenic bacteria such as *Bacteroides*, *Proteus*, *Shigella*, *Erysipelothrix*, and *Streptococcus* increase, while beneficial bacteria such as *Akkermansia*, *Coprococcus*, *Ruminococcus*, and *Blautia* decrease (Liang et al., 2022; Wei et al., 2022). Particularly, SCFA-producing bacteria decrease significantly. Many SCFA-producing bacteria, including *Lachnospira*, *Ruminococcus*, and *Butyricicoccus*, belong to the Firmicutes. Several studies have shown that the ratio of Firmicutes to Bacteroidetes (F/B) is frequently used as an indicator of gut microbiota health (Qiao et al., 2022; Sha et al., 2024). However, this indicator exhibits significant heterogeneity in studies on HUA. Some studies have reported a decrease in the relative abundance of the Firmicutes, an increase in the relative abundance of the Bacteroidetes, and a decrease in the F/B ratio in HUA mice (Guo et al., 2021). Wang et al. (2023a), Wei et al. (2023) also observed similar trends in HUA model mice. In contrast, other studies have reported an increase in the F/B ratio in the HUA group (Li et al., 2022; Zhao S. et al., 2022; Liu et al., 2020). A possible reason for this discrepancy is that the F/B ratio is a macroscopic indicator at the phylum level. An increase in the total abundance of the Firmicutes phylum does not necessarily imply a concurrent increase in beneficial bacteria that produce SCFAs, opportunistic pathogens may also increase. Therefore, particular attention should be paid to the changes in specific functional bacterial genera and their metabolites.

Only one-third of HUA cases progress to gout, an inflammatory and metabolic disorder caused by abnormally elevated uric acid levels and the deposition of urate crystals (monosodium urate, MSU) in joints or surrounding tissues (Thottam et al., 2017). Numerous studies have demonstrated significant differences in gut microbiota among gout patients, asymptomatic patients with HUA, and healthy individuals. Compared with patients with gout, the gut microbiota diversity of asymptomatic HUA individuals is higher, and the ratio of Firmicutes to Bacteroidetes (F/B) is higher, while the ratio of Prevotella to Bacteroides (P/B) is lower (Kim et al., 2022). Healthy individuals demonstrate higher gut microbiota diversity (Shao et al., 2017), with increased abundance of *Bifidobacterium*, *Butyricicoccus*, *Ruminococcaceae*, and *Lachnospiraceae* (Mendez-Salazar et al., 2021). Guo et al.’s (2016) research indicated that gut microbiota in gout patients exhibited abundant levels of *Bacteroides caccae* and *Bacteroides xylanisolvens*, while showing a deficiency in *Faecalibacterium prausnitzii* and *Bifidobacterium pseudocatenulatum*. Chu et al. (2021) conducted metagenomic analyses of healthy individuals and gout patients, revealing increased relative abundances of *Prevotella*, *Fusobacterium*, and *Bacteroides* in gout patients, alongside decreased relative abundances of *Enterobacteriaceae* and butyrate-producing bacteria (Chu et al., 2021). Moreover, studies have shown that from healthy status to asymptomatic HUA and then to gout, the diversity of the gut microbiota exhibits a progressive decline (Wang et al., 2025). Therefore, the distribution of the gut microbiota is significantly correlated with uric acid levels, and gut microbial features are expected to become a new hotspot for predicting disease progression. A recent study found that gut microbial features identified using SHapley Additive exPlanations combined with a Random Forest model can effectively distinguish between gout patients, asymptomatic patients with HUA, and healthy individuals. The model achieved prediction accuracies of 92.46%, 82.35%, and 95.83% when distinguishing healthy individuals from gout patients, healthy individuals from asymptomatic HUA patients, and asymptomatic HUA patients from gout patients, respectively (Tang et al., 2025). This indicates that gut microbial features hold significant potential value in identifying disease progression.

### 4.2 Dysbiosis of the gut microbiota distribution induces inflammation

**FIGURE 2:**
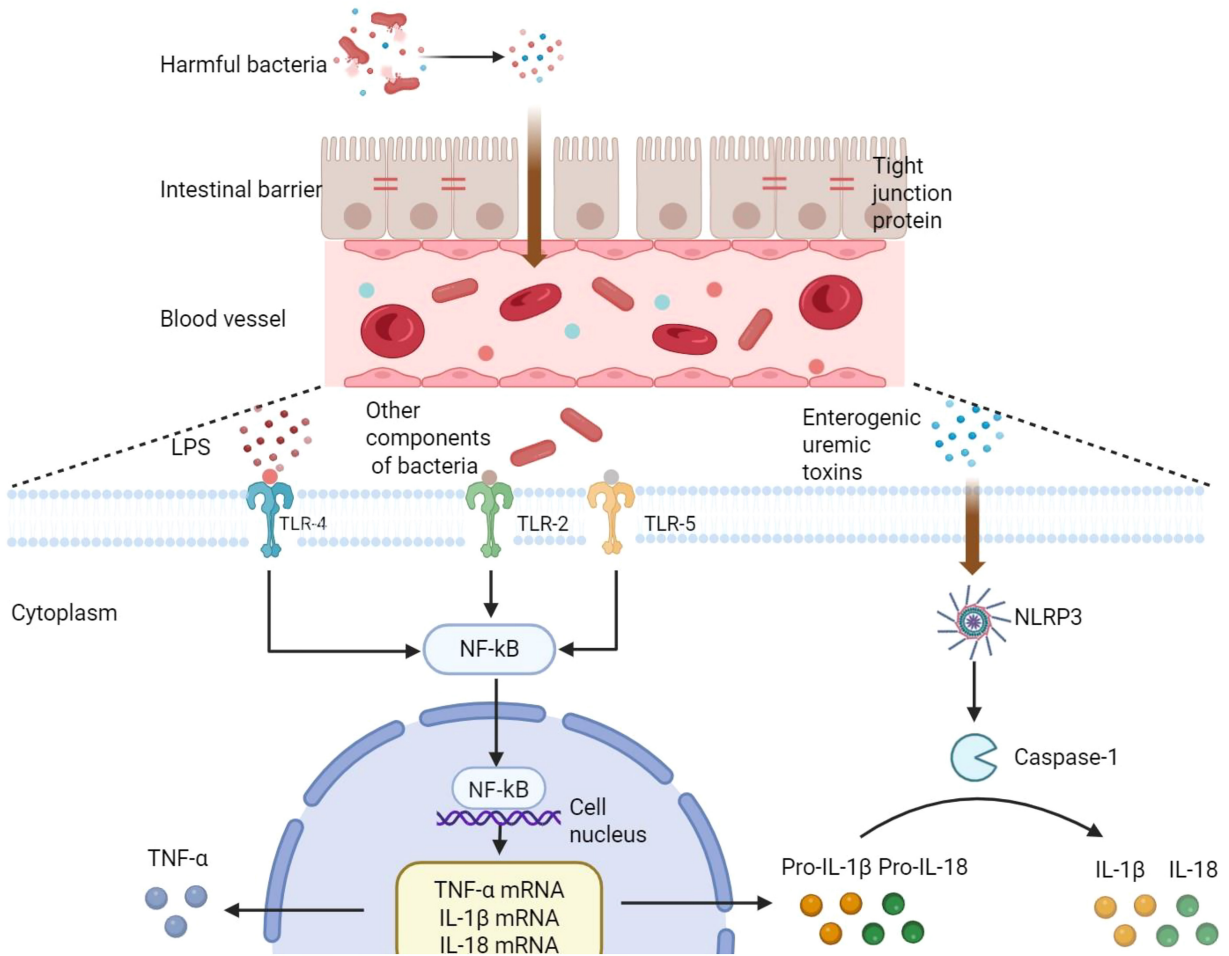
Inflammatory mechanism. An increase in harmful bacteria disrupts tight junction proteins, damaging the intestinal barrier and causing “intestinal leakage”. Intestinal bacteria and their metabolites enter the host, where they are recognized by Toll-like receptors on cell membranes. This triggers the NF-κB pathway in the cell nucleus, producing inflammatory precursor molecules that are released into the cytoplasm. Upon activation of the NLRP3 inflammasome by toxins produced by the gut microbiota, its downstream Caspase-1 effector protein processes the inflammatory precursors into mature inflammatory factors, ultimately resulting in inflammation. Created in BioRender.com.

The integrity of the intestinal barrier is the basis for the excretion of uric acid through the intestine. Its function is to separate the internal environment of the host from the LPS in the intestinal lumen. HUA can activate nicotinamide adenine dinucleotide phosphate (NADPH) oxidase, promoting ROS production that directly damages the integrity of the intestinal epithelium and increases intestinal permeability (Liu P. et al., 2023; Xu et al., 2019). Disruption of the intestinal barrier further induces unrestrained growth of the gut microbiota (Chelakkot et al., 2018). The gut microbiota is a core participant in regulating the intestinal barrier (Yang et al., 2024). Studies indicate that *Ruminiclostridium*, *Bacteroides*, *Akkermansiaceae*, *Bilophila*, *Burkholderiaceae* and *Parasutterella* are key bacteria involved in HUA and compromised intestinal barriers (Lv et al., 2023). HUA patients exhibit reduced levels of beneficial bacteria, decreased tight junction proteins, and increased intestinal permeability. Harmful bacteria proliferate, and the compromised intestinal barrier permits the entry of intestinal bacteria and LPS—a product of the gut microbiota—into the systemic circulation, triggering a systemic inflammatory response known as metabolic endotoxemia (Lv et al., 2020), as shown in Figure 2. This inflammation may arise when Toll-like receptor 4 (TLR4) recognizes LPS from the gut, thereby activating the NF-κB pathway and subsequently promoting the production of inflammatory factors such as TNF-α (Tian et al., 2024). In HUA patients, the abundance of inflammation-associated bacteria increases. Besides LPS, other bacterial components such as flagella, bacterial lipopeptides, lipopolysaccharides, and yeast polysaccharides—acting as ligands—recognize TLR2/4/5 on cell membranes and also promote the release of inflammatory factors like IL-1β and TNF-α (Lv et al., 2020). Research by Zhou et al. (2024) demonstrated that enterogenic uremic toxins produced by the gut microbiota can activate NLRP3 inflammasomes. When NLRP3 inflammasomes are activated by danger signal molecules in the gut, their downstream Caspase-1 effector protein processes inflammatory factor precursors in the cytoplasm into mature inflammatory factors IL-1β and IL-18 (Pan et al., 2022). Then these inflammatory factors are released through the cell membrane into the host’s internal environment, thereby triggering the inflammatory response. Furthermore, the inflammation in the body impairs the normal function of uric acid excretion, ultimately leading to elevated uric acid levels once again. Without intervention, this will lead to a vicious cycle.

## 5 Treatment

Renal excretion disorder and excessive purine metabolism in the liver are the primary mechanisms of elevated blood uric acid. Consequently, the main drugs currently used clinically to reduce uric acid are the XO inhibitors such as allopurinol and the uric acid excretion promoters such as benzbromarone (Yu et al., 2018). However, some patients still experience poor treatment outcomes or cannot tolerate drug side effects. Currently, there are four primary research directions focusing on gut microbiota as a therapeutic target for treating HUA. First, regarding dietary therapy, while traditional low-purine diets can reduce the production of exogenous uric acid, their impact on the gut microbiota structure is limited. In contrast, the Dietary Approaches to Stop Hypertension diet and the Mediterranean diet can effectively increase the abundance of SCFA-producing bacteria and reduce the production of pro-inflammatory metabolites. Recent studies have found that dietary fibers (such as certain coarse grains) (He et al., 2025), polyphenolic compounds (such as dandelion leaf aqueous extract) (Zhou et al., 2025), and protein extracts (such as Leech *Poecilobdella manillensis* protein extract) (Liu X. et al., 2024) can alleviate HUA by regulating the structure or metabolism of the gut microbiota. In terms of clinical trials, two randomized controlled trials have, respectively demonstrated that the Dietary Approaches to Stop Hypertension diet and the Mediterranean diet have a reducing effect on serum uric acid (Gohari et al., 2023; Yokose et al., 2020). Nevertheless, dietary intervention trials currently consist primarily of observational studies, and there is significant heterogeneity among individuals, further verification through large-scale randomized trials is still needed. The next type of intervention treatment is the use of probiotics or prebiotics. Probiotics extensively studied in recent years include *Lactobacillus plantarum* (Fu et al., 2024b), *Lactobacillus rhamnosus* (Fu et al., 2024a), *Bacillus subtilis* (Wang et al., 2023b), and *Lacticaseibacillus paracasei* (Zhou et al., 2026). Certain prebiotics, such as levan (Xu et al., 2025), can increase the abundance of probiotics. A double-blind randomized controlled trial conducted by Lin et al. (2024) showed that serum uric acid levels decreased significantly in all 82 participants after 60 days of combined application of multiple probiotics. However, it should be noted that most of the current evidence stems from small-scale clinical trials and animal experiments, and lacks support from large-scale clinical trials. Differences in probiotic strains and variations in individual gut colonization capacity both limit the reproducibility of therapeutic effects and clinical translation. The third is the intervention treatment with Chinese medicine. In the latest research progress, some traditional Chinese medicines, such as *Cichorium intybus* (Yang et al., 2025), Si Miao San (Qian and Shen, 2023), Smilax glabra Roxb (Xiao et al., 2025), Plantaginis Semen polysaccharides (Zhao et al., 2025), etc., have achieved the goal of reducing uric acid levels by regulating the abundance of gut microbiota. Clinical cohort studies have shown that in 40 patients with HUA treated with Compound Bai Mao Yin, serum uric acid levels decreased by 23.10% after 30 days and by 33.15% after 90 days, through mechanisms such as regulating uric acid transport and improving the gut microbiota (Chen et al., 2025). However, due to the complex composition of traditional Chinese medicine, the specific mechanisms by which it modulates the gut microbiota remain unclear, and the stability and safety of its therapeutic effects require further validation. Finally, there is fecal microbiota transplantation (FMT), which involves transplanting the gut microbiota from a healthy donor into a patient to restore patient’s intestinal microbial balance. In one study, transplanting gut microbiota from young mice to aged mice increased butyrate levels in the aged mice. This cross-age FMT effectively reduced inflammatory responses and improved uric acid metabolism (Song et al., 2025). Further studies indicated that FMT not only reduced uric acid levels but also restored gut microbiota balance (Yuan et al., 2025). In addition, a retrospective clinical trial analyzed uric acid levels in 144 patients before and after FMT and found that washed microbiota transplantation can reduce serum uric acid levels in patients with HUA in the short term (Cai et al., 2022). However, FMT carries potential safety risks. A study summarizing adverse events associated with FMT reported over a 20-year period found that 19% of patients experienced such adverse events, including diarrhea, abdominal pain, and infections, suggesting that this treatment should be used with strict caution (Marcella et al., 2021). In summary, while HUA treatment strategies targeting the gut microbiota have a broad application prospect, current research is still limited to animal experiments and preliminary clinical trials. The efficacy, safety, and individual applicability of these various approaches require verification through large-scale and standardized clinical studies.

## 6 Summary and outlook

This paper provides a systematic review of the relationship between gut microbiota and HUA. On one hand, the gut microbiota influences uric acid production by participating in purine metabolism. Its metabolite uricase contributes to uric acid breakdown, amino acids affect uric acid levels, and SCFAs influence uric acid excretion. On the other hand, elevated uric acid levels in HUA patients disrupt the distribution of gut microbiota, leading to impaired intestinal barrier function and thereby inducing systemic inflammation and localized chronic inflammation in the gut. This clarification of the correlation breaks through the previous traditional understanding that only focused on hepatic production and renal excretion of uric acid, providing a novel basis for the prevention and treatment of HUA. Future research should delve deeper into the specific molecular mechanisms and signaling pathways based on gut microbiota characteristics. It should also combine novel therapeutic strategies such as dietary therapy, probiotics, prebiotics, Chinese medicine, and FMT with traditional treatment methods, thereby offering patients a more diverse range of therapeutic options.
